# Enhancing socio-emotional learning and mental health through computational thinking: a cross-cultural analysis of the COMPUSEL programme

**DOI:** 10.3389/fpsyg.2025.1480731

**Published:** 2025-04-25

**Authors:** Adelinda Candeias, Adriana Felix, Anisoara Dumitrache, Beatrice Almășan, Ilke Evin Gencel, Magdalena Zadworna, Karolina Kossakowska, Angeliki Sakellariou, Miltos Sakellariou, Cavus Sahin, Cigdem Sahin Taskin

**Affiliations:** ^1^School of Health and Human Development, Comprehensive Health Research Centre, University of Evora, Évora, Portugal; ^2^Faculty of Psychology and Educational Sciences, University of Bucharest, Bucharest, Romania; ^3^Faculty of Education, Izmir Demokrasi University, Izmir, Türkiye; ^4^Faculty of Educational Sciences, Institute of Psychology, University of Lodz, Lodz, Poland; ^5^Social Cooperative of Cyclades, Syros, Greece; ^6^Faculty of Education, Canakkale Onsekiz Mart University, Canakkale, Türkiye

**Keywords:** social-emotional learning, computational thinking, mental health, digital stories, educational intervention

## Abstract

**Purpose:**

The COMPUSEL curriculum is designed to enhance primary school students’ five core Socio-emotional (SEL) competencies (self-awareness, self-management, social awareness, relationship skills, and responsible decision-making) and mental health by incorporating computational thinking. This study evaluates the curriculum’s impact across five European countries (Greece, Poland, Portugal, Romania, and Turkey), analyzing cultural differences in its implementation.

**Methodology:**

A quasi-experimental research design was employed to examine the curriculum’s impact. The piloting process included a training seminar for teachers, after which a volunteer teacher was selected to implement the curriculum. The curriculum featured digital stories and a comprehensive guide for teachers, which outlines the COMPUSEL learning model and provides guidance for effective implementation. Data were collected from 113 students through pre-test and post-test evaluations, without the use of a control group.

**Findings:**

The findings revealed statistically significant differences across all dimensions in Greece, Turkey, and Poland. In Portugal, significant differences were noted in four dimensions, with the exception of responsible decision-making. Conversely, no statistically significant differences were observed in any dimension in Romania.

**Conclusion:**

The COMPUSEL project’s curriculum is designed to streamline planning and offer flexible models that promote sustainable education, catering to diverse student needs. By integrating digital storytelling and computational thinking into SEL, the curriculum provides a holistic approach that equips students with essential 21st-century life skills. The study also highlighted the varied cultural effects on the curriculum’s implementation across different countries. These cultural variations may have influenced the demonstration of the curriculum’s overall effectiveness, yet the research confirms that the COMPUSEL curriculum is effective in most participating countries.

## 1 Introduction

The importance of social-emotional learning (SEL) competencies is widely acknowledged for cultivating effective students, citizens, and workers (Weissberg and Cascarino, 2013). Numerous school-aged children in Europe face social–emotional difficulties (Cefai et al., 2018). Prominent international organizations such as UNESCO, UNICEF, OECD, and WHO have issued guidelines to effectively address these issues. Consequently, the focus on SEL is increasing in Europe and globally. It is progressively seen as an essential 21st-century skill, becoming a key component of educational curricula across European nations (Cefai et al., 2018; OECD, 2021). Dracinschi (2012) noted that SEL issues are integrated into European curricula.

Evidence is mounting that universal interventions designed to enhance mental health and wellbeing through educational and systemic changes are effective in supporting the mental health and wellbeing of young people (Durlak et al., 2011; Weare and Nind, 2011; Franco et al., 2017; Goldberg et al., 2019; OECD, 2021). A comprehensive review of over 200 studies on SEL in schools by Durlak et al. (2011) found that universal SEL interventions improved social and emotional skills, fostered positive attitudes, encouraged prosocial behavior, boosted academic performance, and reduced mental health issues such as antisocial behavior, substance abuse, anxiety, and depression. Further research underscores the significance of school-based mental health interventions as a preventive measure against the development of mental health problems during adolescence (Candeias and Félix, 2023; Candeias et al., 2024a,b; Stockings et al., 2016; Woods and Pooley, 2016; Currie and Morgan, 2020). An OECD report examining SEL interventions in schools across ten major global cities revealed a strong correlation between these interventions and both psychological wellbeing and academic success among children aged 11 and 15. The effectiveness of these interventions was particularly linked to their ability to influence behavior, lifestyles, and cognitive skills (OECD, 2021).

In parallel, sustainability education has gained increasing attention as an essential aspect of high-quality education. Rooted in early efforts to address environmental challenges, sustainability education aims to equip students with the knowledge, skills, values, and competencies necessary to understand and respond to global issues (Grange, 2017; Velasco, 2021). The 2030 Agenda for Sustainable Development highlights the need to integrate education for sustainable development (ESD) at all levels, from early childhood education to higher education, as well as within non-formal and informal learning contexts. ESD fosters competencies related to sustainability, addressing themes such as global inequalities in resource access, economic development, justice, peace and conflict resolution, human rights, and ecosystem preservation (Wals and Jickling, 2002). Recent research has emphasized the importance of equipping both educators and students with sustainability-related skills through transdisciplinary pedagogical approaches that promote self-directed learning, collaboration, and problem-solving (Bürgener and Barth, 2018; Cebrián et al., 2020; Mulà et al., 2017; Rieckman, 2018).

This study investigates the necessity of a SEL program that incorporates computational thinking skills to enhance SEL in primary school students. It first examines the current state and evolution of SEL in education and the challenges in promoting it. Additionally, the study explores the potential of computational thinking to support SEL. The combination of Social-Emotional Learning (SEL) with Computational Thinking enables the structured development of emotional and social skills, fostering problem-solving, decision-making, and emotional regulation through logical and analytical processes, while its application in a multicultural approach allows for testing the intervention’s effectiveness across different countries, respecting and adapting to their cultural and educational specificities. Based on this analysis, the study presents the COMPUSEL project, an ERASMUS+ initiative (2021-1-TR01-KA220-SCH-000031609), which aims to merge social–emotional development with computational thinking to enhance problem-solving skills and deepen students’ understanding of their own and others’ emotions.

### 1.1 SEL

SEL is a comprehensive approach that fosters the acquisition of essential life skills such as emotional awareness, empathy, and responsible decision-making. These competencies are instrumental in guiding individuals’ thoughts, feelings, and behaviors, ultimately contributing to success in educational settings and throughout life [Collaborative for Academic, Social, and Emotional Learning (CASEL, 2003, 2012, 2013, 2016, 2020)]. SEL programmes vary widely but generally emphasize cognitive regulation and executive functioning skills that are critical for mental processes involved in focusing, planning, and controlling behaviors (Jones et al., 2018; Jones and Kahn, 2017; Jones et al., 2016). According to CASEL (2013) SEL helps individuals develop key skills, including the ability to understand and manage emotions, set and achieve positive goals, feel and show empathy for others, establish and maintain positive relationships, and make responsible decisions. These skills are encapsulated in the five core competencies of SEL: self-awareness, self-management, social awareness, relationship skills, and responsible decision-making.

Research supports the beneficial impact of SEL on various aspects of students’ lives, including academic performance, social behaviors, and mental health outcomes. SEL not only improves individual student outcomes but also contributes to a positive school climate and enhances interpersonal relationships among students and staff (Zins and Elias, 2007; Durlak et al., 2011).

Globally, the integration of SEL into education systems varies, with countries adopting different frameworks and curricula to enhance students’ social and emotional competencies. In Europe, two main approaches to implementing SEL have emerged: adopting existing interventions developed in other countries or designing and implementing original SEL programmes tailored to national contexts (Humphrey, 2018; Sahin Taskin, 2021). For example, in Romania, SEL is not part of a dedicated curriculum but is embedded within primary education subjects such as Personal Development, with increasing awareness and initiatives promoting SEL at both macro and micro levels. Greece integrates SEL through Health Education programmes, implemented nationwide since 1996, emphasizing mental and social well-being, critical thinking, and social skills, though participation remains voluntary. Turkey has progressively recognized the importance of SEL (Isik and Sahin-Taskin, 2024; Korkmaz and Güney Karaman, 2024) by incorporating it into preschool and primary school curricula, with programmes such as the Training Program in Primary Schools (IYEP) and the Classroom Guidance Program, in collaboration with UNICEF (MoNE, 2020). However, challenges remain, including limited awareness among educators and parents. Poland mandates SEL development through School Prevention Programmes, ensuring psychosocial skills training across all schools (Kwiatkowski, 2016; Barabasz, 2021; Skibińska and Zacniewska, 2021), with additional support from non-governmental organizations like the Center for Citizenship Education (CCE) (Gaś, 2006; Kossakowska and Zadworna, 2021). Portugal has made progressive strides in SEL integration through policy changes, including the “Profile of students leaving compulsory education” and the optional Education for Citizenship subject, allowing flexibility in addressing socio-emotional needs (Candeias et al., 2024a,b; Costa and Faria, 2013; Matos et al., 2022; Cristóvão et al., 2017).

Based on the analysis of SEL implementation in the partner countries, several key aspects stand out:

- Across all countries, SEL is recognized as an important component of education, and significant efforts have been made to integrate it into national curricula.
- Policymakers prioritize SEL development, acknowledging its role in fostering students’ social and emotional competencies.
- While none of the partner countries have a fully dedicated SEL curriculum, SEL skills are incorporated into various subjects at different educational levels.
- Teachers play a central role in implementing SEL, underscoring the need for continuous training and institutional support.
- Educational systems across these countries emphasize values such as personal development, active citizenship, and social inclusion.
- The development and implementation of SEL are influenced by various factors, including national educational policies, the overall organization of the education system, school autonomy, and resource availability.

Despite these differences, all five countries acknowledge the significance of SEL and continue to develop policies, programmes, and collaborations, such as Erasmus + projects, to strengthen students’ emotional and social well-being.

In conclusion, as the educational landscape evolves, SEL remains a fundamental component of comprehensive education reforms aimed at preparing students for academic and life success. The ongoing development and integration of SEL across different educational systems highlight its crucial role in fostering students’ social, emotional, and academic growth. By prioritizing SEL, education systems can better equip students with the necessary skills to navigate increasingly complex social and professional environments (CASEL, 2003, 2012, 2013, 2016).

### 1.2 Computational thinking and SEL

Computational thinking is considered a vital 21st-century skill (Haseski et al., 2018; Rich et al., 2019). Many researchers argue that it should be developed in students of all ages to equip them with the skills needed for various applications, including social and interpersonal skills (Hsu et al., 2018; Lockwood and Mooney, 2018; Wing, 2006). This is where Social-Emotional Learning (SEL) becomes relevant, focusing on empathy, mindfulness, relationship skills, and responsible decision-making.

Computational thinking can be integrated into SEL in the following ways in the decomposition phase (Futschek, 2006), outlines the steps and rules necessary to achieve a desired outcome (Lamagna, 2015). Algorithms, therefore, represent step-by-step solutions to specific problems, making the solutions more manageable. Computational thinking is the process of organizing effective thinking, crucial for identifying and solving problems encountered in daily life (Wing, 2006, 2011). The four stages of computational thinking are essential for organizing effective thinking processes. This skill is critical for identifying and solving daily life problems: Decomposition (Denning and Tedre, 2019), it involves dividing a task into smaller elements, each of which can be efficiently handled. Abstraction: It involves highlighting essential details and suppressing non-essential ones (Wing, 2008, 2011). During abstraction, attention is given solely to the critical aspects of a problem or context (Washington et al., 2021); Pattern Recognition: involves identifying recurring situations and generalizing them (Rodríguez del Rey et al., 2021); Algorithmic Thinking automates the problem-solving process through a series of systematic logical steps (Lamagna, 2015). These algorithms serve as formulas for calculating answers, processing data, or automating tasks.

Integrating computational thinking into SEL can be highly advantageous as it provides a structured approach to problem-solving that can be applied to social and emotional challenges. Although there is no standardized methodology for SEL, using the four computational thinking stages can effectively develop SEL competencies. For example, by recognizing patterns of empathic behavior, students can learn appropriate and supportive responses when peers face challenges. SEL and computational thinking share the common goals of fostering critical thinking, problem-solving, and decision-making skills.

To summarize, we could observe that each country’s approach to SEL reflects its cultural, educational, and policy contexts, demonstrating a shared recognition of the importance of SEL while also highlighting diverse implementation methods. Schools play a crucial role in fostering both SEL and computational thinking. By incorporating universal interventions based on innovative curricula and materials, schools can support teachers in effectively intervening with all students.

The purpose of this study aims to evaluate the effects of a SEL program that integrates computational thinking skills to enhance SEL among primary school students. The program includes innovative curriculum materials designed to incorporate computational thinking skills, featuring training resources such as creative digital stories and a teacher handbook to support professional development. The study examines the competencies of students before and after the intervention, who completed the program as part of their mainstream curriculum over a 10-week period. The program aimed to enhance social and emotional competence through computational thinking and digital stories. The assessment is based on teachers’ evaluations. It was hypothesized that students who participated in the COMPUSEL program would exhibit higher levels of social and emotional competencies, including self-awareness and management, social awareness, relationship skills, and decision-making skills, as well as improved prosocial behavior.

The expected outcomes, the COMPUSEL programme aims to improve students’ well-being, enhance their socio-emotional competencies, and demonstrate the positive impact of computational thinking on learning. By providing a structured framework for SEL, the programme supports students in developing essential life skills, fostering emotional regulation, and improving decision-making abilities.

## 2 Method

A quasi-experimental longitudinal design was employed to evaluate the impact of the Compusel program on students’ outcomes by comparing the results within time points (pre-test vs. post-test) without a control group. This design was chosen due to the practical constraints of educational interventions in real-world school settings. Given the ethical considerations and logistical challenges of withholding an educational intervention from a control group, the study instead focused on a pre-test/post-test comparison to assess changes over time. This design allows for evaluating the impact of COMPUSEL while ensuring all participating students benefit from the intervention. Although the absence of a control group limits causal inferences, the study could provide valuable insights into the effectiveness of SEL and computational thinking in diverse cultural contexts.

The project involved five implementation countries— Greece, Poland, Portugal, Romania and Turkey—each of which collected data in their respective languages. All data were compiled into a central database using Google Drive and Excel files.

### 2.1 The intervention

The Compusel program is a comprehensive intervention designed to emphasize the importance of collaboration among students, teachers, school leaders, community stakeholders, and policymakers. The program focuses on three key domains: the promotion of SEL, the enhancement of computational thinking, and the promotion of social, emotional, and behavioral competencies. Each domain is divided into specific topics, with the SEL component covering five main areas: self-awareness, self-management, social awareness, relationship skills, and responsible decision-making; and the computational thinking covering the stages: decomposition, abstraction, pattern recognition, and algorithmic thinking.

The piloting phase of the COMPUSEL curriculum involved several key components: training courses and supervision for teachers, manualized handbooks and an app with digital stories and guidelines, and meetings with school leaders and teachers. The process began with a training seminar, followed by selecting a volunteer teacher in each country to implement the curriculum. A training support team was established in each country to coordinate training activities, supervise teachers, and adapt materials. After obtaining ethical approval, the curriculum was implemented in the spring semester of 2024. Project staff conducted classroom visits and informal discussions with teachers to address issues. Pupils completed the Social and Emotional Questionnaire (SEQ) before and after implementation, and a semi-structured interview, the Quality assessment interview (QAI), was conducted with the classroom teacher for feedback.

#### 2.1.1 Training courses and supervision

Teachers participated in initial training (between 8 and 16 h) that focused on practical and theoretical knowledge for promoting SEL and computational thinking in the school context. The training provided tools and materials for implementing these activities and included guidance on using the provided handbook and curriculum, as well as utilizing digital stories and activities designed to explore these stories through the stages of computational thinking. The training sessions were conducted either in person or remotely. During the implementation the national teams maintain the contact with teachers. After the implementation quality assurance procedures were in place to monitor the quality of the program’s implementation across various schools and countries.

#### 2.1.2 Handbook and digital stories activities and guidelines

The Compusel curriculum includes a comprehensive handbook that outlines the curriculum and provides guidelines for step-by-step activities for students aged 8–12 and their teachers, to be integrated into the mainstream curriculum at school. Additionally, the curriculum features ten Digital Stories, with two stories dedicated to each domain of SEL, as well as an Activity Book ^1^that complements the digital content. All materials for teachers and students have been adapted and translated into the languages of the five countries participating in the experimentation (Greek, Polish, Portuguese, Romanian, and Turkish), as well as English to facilitate broader accessibility and collaboration among international partners.

#### 2.1.3 SEL curriculum for primary education

The curriculum ^2^employs computational thinking as a pedagogical approach and includes five modules that align with CASEL’s SEL components and incorporates a variety of activities and strategies to develop competencies related to self-awareness, self-regulation, social awareness, relationship building, and responsible decision-making. The primary goal is to internalize social–emotional skills and enhance the well-being of primary students. The curriculum is tailored to students’ developmental stages and includes engaging and interactive activities such as role-playing, cooperative projects, group discussions, and digital stories. These activities help students apply SEL skills in real-life situations, promoting a deeper understanding and practical application.

#### 2.1.4 Digital stories

The COMPUSEL project integrates Digital Stories into its curriculum to improve students’ socio-emotional skills and mental health (Alismail, 2015). These stories are crafted to be relatable and realistic, depicting situations that students commonly face, such as bullying or peer pressure. The goal is to enhance critical skills like empathy, problem-solving, and responsible decision-making by engaging students with scenarios that require them to apply computational thinking.

The curriculum encourages teachers to use these stories as a tool to facilitate discussion and reflection, reinforcing the lessons learned and helping students apply these skills in their own lives. The alignment of digital stories with specific learning objectives ensures that the content is not only engaging but also educationally effective, promoting a holistic approach to student well-being and academic success.

Each story immerses students in a narrative that promotes the development of self-awareness, self-management, social awareness, relational skills, and responsible decision-making. These stories incorporate computational thinking steps such as decomposition, pattern recognition, abstraction, and algorithmic thinking to enhance both cognitive and emotional skills.^3^

#### 2.1.5 Meetings with schools

To support the systemic implementation of the Compusel curriculum, a total of at least 4 h, spread over 2 meetings, were conducted for school leaders and teachers. These meetings have been held monthly, beginning in September 2023 and during intervention (between March and June 2024).

### 2.2 Participants

The study included a total of 113 primary school pupils (50 females and 63 males) from 6 classrooms in 7 schools across five European countries (see Table 1). The participants were drawn from grades 3 to 5, representing children aged approximately 8 to 12 years old. However, it is important to note that the structure of primary education varies across the participating countries, with differences in duration and age range. The sample included a mix of public and private schools located in both urban and rural areas, ensuring a diverse representation of different educational contexts. Additionally, six teachers participated in the study. It is important to note that these teachers had varying levels of experience with SEL and computational thinking. While some had prior exposure to SEL concepts through professional development initiatives, others were newly introduced to these methodologies as part of the project. Similarly, computational thinking was a relatively new concept for most of the educators, requiring additional training and adaptation to the COMPUSEL curriculum. The teachers received training on theoretical concepts and used the digital stories, the handbook, and the activities provided by the project (previously mentioned materials). Ethical approval was granted by academic institutions and educational authorities, and legal representatives of all the participants provided consent. Participation was voluntary, with no financial incentives offered.

**Table 1:** Gender distribution and mean age of participants by country.

		Gender		Age mean
		Boy	Girl	
Country	Portugal	9	9	9.94
	Greece	13	17	12
	Poland	13	10	10
	Romania	13	5	11
	Turkey	15	9	9.75

### 2.3 Measurement tools

Social and Emotional Questionnaire (SEQ): This tool, created by Sahin Taskin (2024) and was translated and adapted for English, Greek, Polish, Romanian and Turkish by the network of COMPUSEL. SEQ measures the social and emotional learning (SEL) of pupils aged 9–12 years, completed by teachers. It is based on five SEL domains: self-awareness, self-management, social awareness, relationship skills, and responsible decision-making. The SEQ comprises 32 items, with each subscale corresponding to one of the five SEL: Self-Awareness has 6 items, Self-Management has 6 items, Social Awareness has 7 items, Relationship Skills has 8 items, and Responsible Decision Making has 5 items. An example item from the Social Awareness subscale is “I understand other people’s feelings” or “I am aware of how I behave in the face of events.” The SEQ demonstrates strong reliability The study utilized Cronbach’s alpha of 0.960 for the composite score and Cronbach’s alpha between 0.838 and 0.884 for the five subscales (Anastasi and Urbina, 1988). An Exploratory Factor Analysis (EFA) was conducted using the Principal Component Method with orthogonal (varimax) rotation (KMO = 0.918). Based on the subscales of the questionnaire, the factor analysis was forced to 5 factors, which accounted for 63.854% of the variance in accordance with previous studies (Sahin Taskin, 2024). Both the composite score and the individual subscales were employed in this research.

Quality assessment interview (QAI): Teachers also answer an interview with six questions examining the role of computational thinking in teaching SEL competencies, especially the impact of digital stories on student engagement and motivation, challenges faced, and suggestions for improvement. The QAI was translated and adapted for english, greek, polish, romanian and turkish. And content analysis was made for 2 team members in each country with an agreement of at least 80%.

The questionnaire included a number of demographic questions about the students’ age, school level, and gender.

### 2.4 Procedures

The COMPUSEL program was implemented in selected schools based on their willingness to participate and their socioeconomic and geographic diversity. Teachers were chosen for their expertise in SEL, while students were included based on their enrolment in the targeted grade levels. To ensure effective implementation, teachers underwent blended training (online and face-to-face), covering SEL principles, computational thinking, and digital storytelling, with access to a manual and digital resources. The program was integrated into classrooms through structured activities that fostered problem-solving, emotional regulation, and collaboration, reinforced by discussions and adaptations to each school’s schedule.

Assessment involved pre- and post-program SEL evaluations using standardized instruments, along with teacher interviews and classroom observations for qualitative insights. Data confidentiality was ensured through anonymization and secure storage, with informed consent obtained from all participants. Each country had a national coordinator overseeing implementation and data collection, supported by regular virtual meetings to maintain alignment across contexts. The study followed the Declaration of Helsinki (2013), with ethical approval and voluntary participation ensuring data protection and research integrity.

## 3 Data analysis

Data were analyzed using IBM SPSS version 28. Students were matched using a code to integrate their pretest and post-test scores, ensuring that only those with scores from both tests were included in the dataset. Missing values were imputed with the mean score of the corresponding test item. The Kolmogorov–Smirnov test was employed to examine the distribution shape of scale’s scores and we observe that the criteria of normality assumption was satisfied (*D* = 0.984, *p* = 0.763) (Alvo and Yu, 2018).

A paired t-test was employed to understand whether there is a difference in students’ responses between pre-test and post-test. Effect sizes were estimated and interpreted using Cohen’s d (*d* > 0.2) indicating a small effect, *d* > 0.5 a moderate effect, and *d* > 0.8 a large effect (Cohen, 1988).

To analyze teachers’ perceptions, we use a qualitative analysis, based on the contents of the discourse.

## 4 Results

### 4.1 Analysis of the effects of a social-emotional learning (SEL) program among primary school students

The study assessed the effectiveness of an SEL intervention by measuring changes in self-awareness, self-management, social awareness, relational skills, and responsible decision-making across a pre- and post-test setup. The global analysis, encompassing all participants (*N* = 113), revealed statistically significant improvements in all components. Specifically, significant enhancements were noted in self-awareness (t (88) = −9.623, *p* < 0.001, *d* = 3.293), self-management (*t* (88) = −7.932, *p* < 0.001, *d* = 3.808), social awareness (*t* (88) = −8.229, *p* < 0.001, *d* = 3.877), and relational skills (*t* (88) = −7.571, *p* < 0.001, *d* = 4.620), responsible decision-making (*t* (88) = −7.571, *p* < 0.001, *d* = 4.620) (Table 2).

**Table 2:** Paired samples statistics and t-tests for SEL components (*N* = 113).

Country	Subscale	1st Moment M (SD)	2nd Moment M (SD)	*t*	*df*	*p*	d de Cohen
GR	Self- awareness	12.100 (2.670)	14.970 (2.220)	−9.255	29	<0.001	1.697
	Self- management	11.130 (3.550)	13.700 (2.938)	−6.929	29	<0.001	2.029
	Social awareness	17.870 (2.209)	20.030 (1.450)	−6.517	29	<0.001	1.821
	Relational skills	17.070 (3.619)	18.900 (3.325)	−5.350	29	<0.001	1.877
	Responsible decision-making	9.630 (2.684)	11.670 (2.023)	−5.969	29	<0.001	1.866
	Total scale	67.800 (11.929)	79.267 (9.545)	−10.603	29	<0.001	5.923
PL	Self- awareness	8.960 (1.551)	15.430 (1.727)	−14.307	22	<0.001	2.172
	Self- management	9.090 (1.564)	16.390 (1.751)	−14.823	22	<0.001	2.363
	Social awareness	11.960 (2.549)	19.520 (1.592)	−11.809	22	<0.001	3.072
	Relational skills	12.870 (2.341)	22.350 (1.921)	−14.652	22	<0.001	3.102
	Responsible decision-making	7.960 (2.056)	13.430 (1.376)	−10.070	22	<0.001	2.609
	Total scale	50.826 (5.087)	87.130 (5.675)	−23.546	22	<0.001	7.394
PT	Self- awareness	14.110 (3.939)	16.940 (1.434)	−3.157	17	0.006	3.808
	Self- management	12.500 (3.365)	15.000 (1.645)	−3.058	17	0.007	3.468
	Social awareness	15.890 (3.411)	18.890 (2.055)	−3.674	17	0.002	3.464
	Relational skills	18.500 (3.276)	21.060 (1.626)	−3.142	17	0.006	3.451
	Responsible decision-making	13.220 (1.665)	13.560 (1.338)	−0.825	17	0.421	1.715
	Total scale	74.222 (12.896)	85.444 (6.419)	−3.635	17	0.002	13.099
RO	Self- awareness	15.670 (2.787)	16.390 (1.461)	−1.000	17	0.331	3.064
	Self- management	14.500 (2.572)	14.220 (2.074)	0.341	17	0.738	3.461
	Social awareness	18.440 (3.166)	18.890 (1.568)	-0.536	17	0.599	3.518
	Relational skills	20.110 (3.563)	20.720 (1.674)	−0.623	17	0.541	4.161
	Responsible decision-making	12.610 (2.253)	12.670 (1.572)	−0.081	17	0.936	2.900
	Total scale	81.333 (13.262)	82.889 (6.125)	−0.412	17	0.685	16.012
TR	Self- awareness	13.710 (4.154)	16.210 (1.215)	−3.283	23	0.003	3.730
	Self- management	12.830 (3.158)	14.960 (1.829)	−0.748	23	0.004	3.261
	Social awareness	16.210 (4.872)	19.330 (1.435)	−1.139	23	0.003	4.703
	Relational skills	17.040 (4.448)	20.630 (1.996)	−1.671	23	<0.001	4.529
	Responsible decision-making	11.960 (2.836)	13.330 (1.685)	−0.270	23	0.017	2.618
	Total scale	71.750 (18.164)	84.458 (5.890)	−3.671	23	0.001	16.959
All sample	Self- awareness	12.690 (3.785)	15.870 (1.820)	−9.957	112	<0.001	3.392
	Self- management	11.830 (3.409)	14.810 (2.345)	−8.516	112	<0.001	2.712
	Social awareness	16.090 (3.995)	19.420 (1.1635)	−8.744	112	<0.001	4.045
	Relational skills	16.920 (4.184)	20.600 (2.583)	−8.542	112	<0.001	4.581
	Responsible decision-making	10.830 (3.053)	12.840 (1.801)	−7.122	112	<0.001	2.998
	Total scale	68.363 (16.121)	83.531 (7.574)	−9.762	112	<0.001	16.518

The study analyzed the effectiveness of SEL interventions across Greece, Poland, Portugal, Romania, and Turkey, revealing varied cultural impacts. In Greece, significant improvements were observed in self-awareness (*d* = 1.697) and self-management (*d* = 2.029). Poland showed the most considerable gains, especially in relational skills (*d* = 3.102) and self-management (*d* = 2.363). Portugal saw high improvements in self-awareness and self-management, though responsible decision-making did not significantly improve. Romania’s results were mixed, with minor improvements in self-awareness (*d* = 3.064). In Turkey, moderate improvements were noted in relational skills (*d* = 4.529) and social awareness (*d* = 4.703). These findings highlight the varying efficacy of SEL interventions across different cultural contexts, suggesting that cultural adaptations of SEL programmes may be beneficial to maximize their efficacy.

### 4.2 Analysis of quality of the curriculum using computational thinking in teaching SEL competencies, especially the impact of digital stories

Additionally, the results from the Quality assessment interview (QAI), where teacher provided qualitative insights into the implementation and impact of the COMPUSEL program, namely the role of computational thinking in teaching SEL competencies, especially the impact of digital stories on student engagement and motivation, challenges faced, and suggestions for improvement. Examples of representative responses from 5 countries are presented in Table 3, based on exploratory content analysis, that use complete sentences.

**Table 3:** Content analysis from teacher interviews (*N* = 6).

Topic	Summary of responses
Contribution of computational thinking to teaching social–emotional skills	*Teachers find that using computational thinking (CT) to teach social competences is beneficial because it helps students analyze emotional situations, draw on past experiences, and apply learned strategies to new scenarios.* *CT enhances problem-solving skills crucial for emotional control and decision-making by breaking down complex issues into simpler components.* *This approach boosts both technical and emotional competencies. Social-emotional learning (SEL) also aids in self-understanding, making problem-solving easier and enabling children to manage and resolve challenges effectively.*
Students’ approach to problem-solving after COMPUSEL implementation	*The introduction of the COMPUSEL program has significantly enhanced students’ problem-solving methods, making them more efficient and collaborative, and better prepared to tackle academic and real-life challenges.* *In my classroom, the program has effectively addressed students’ issues, especially in anger management. Students are gaining self-awareness and frequently inquire about their progress. A recent survey highlighted this growth, with one student noting a significant improvement in conflict resolution and communication skills compared to last year, which is very encouraging.* *The students started applying decomposition and abstraction to resolve conflicts.*
Role of digital stories in developing social–emotional competencies	*Digital stories are essential in education, creating an immersive and dynamic learning environment that fosters empathy, conflict resolution, teamwork, emotional expression, critical thinking, and creativity.* *In my classroom, after listening to digital stories, we discuss whether students have encountered similar situations. Many have, such as two students who recognized their own past mistakes through a recent story. Despite needing improvements in audio quality and engagement, the stories effectively facilitate these realizations.*
Increase in student interest and motivation during COMPUSEL implementation	*Students’ interest and motivation improved due to the engaging and relevant digital stories and panel discussions used in the sessions.* *The digital stories have boosted student motivation, particularly in fostering responsibility. Students relate to characters in the stories, using them as examples in peer discussions.*
Challenges in implementing COMPUSEL	*Despite initial challenges in implementing the program, these were successfully resolved. Future recommendations include continuous training for facilitators, regular program evaluations, and exploring alternative assessment tools to more accurately measure the program’s effectiveness.* *The time for implementation was too short, distributing the program throughout the year would enhance its effectiveness. Implementation faced challenges, however, we managed to maintain motivation and encountered no significant issues.* *Teachers appreciated the support during the process, with “extremely useful resources” and examples on how different learning activities can be created and approached.*

The qualitative findings, based on teachers’ narratives, support the quantitative results, highlighting the benefits of integrating computational thinking into SEL through the COMPUSEL curriculum. Teachers observed that computational thinking enhances students’ emotional and technical skills, improving problem-solving, self-awareness, conflict resolution, and communication. The curriculum has notably improved students’ problem-solving methods, making them more efficient, collaborative, and better prepared for academic and real-life challenges. It effectively addressed issues like anger management, with students gaining self-awareness and showing significant improvements in conflict resolution and communication skills.

Digital stories played a key role in fostering empathy, critical thinking, and creativity, though improvements in engagement and audio quality are needed. They have been crucial in creating an immersive learning environment that fosters empathy, conflict resolution, teamwork, emotional expression, critical thinking, and creativity. These stories help students relate to and learn from characters’ experiences, though improvements in engagement and audio quality are needed. The curriculum increased student motivation and responsibility. Despite initial challenges, these were resolved. Future recommendations include ongoing facilitator training, regular evaluations, spreading the curriculum throughout the academic year for greater impact and exploring alternative assessment tools to better measure effectiveness.

## 5 Discussion

Primary school education lays the foundation for students’ future development, and integrating social-emotional learning (SEL) at an early stage is essential (Candeias et al., 2024a,b; Galindo et al., 2022; Sahin Taskin, 2021). The COMPUSEL program demonstrated notable improvements in SEL across different countries, though with varying results.

Greece showed significant gains in self-awareness and self-management, indicating that students became more reflective and emotionally aware. In Poland, relational skills and self-management improved, reflecting a strong emphasis on collaboration. Turkey saw growth in social awareness and relational skills, aligning with national SEL policies (MoNE, 2020). Portugal showed progress in most dimensions except responsible decision-making, suggesting a need for further pedagogical support. Romania, however, exhibited no statistically significant changes, likely due to policy and implementation challenges (Kossakowska and Zadworna, 2021).

The integration of computational thinking within SEL instruction provides a structured and systematic method for addressing emotional and social challenges, allowing students to break down complex emotions into smaller, more manageable parts through decomposition, abstraction, pattern recognition, and algorithmic thinking (Denning and Tedre, 2019; Futschek, 2006). The results from teacher interviews reinforce this, with educators highlighting that computational thinking helps students develop problem-solving skills, emotional regulation, and collaborative decision-making. Teachers also noted that students applied computational thinking principles when resolving interpersonal conflicts, enhancing their ability to analyze emotional situations and make informed decisions.

A key component of the COMPUSEL program is the use of digital storytelling to teach SEL skills. Digital stories proved effective in enhancing empathy, critical thinking, and creativity, as they allowed students to engage with realistic and emotionally complex narratives. However, teachers also identified areas for improvement, such as enhancing the audio quality and engagement level of digital materials. The use of digital storytelling aligns with previous studies highlighting its effectiveness in fostering self-awareness, relational skills, and social–emotional reflection (Hwang et al., 2014; Cristóvão et al., 2017; Gigantesco et al., 2019; Smeda, et al., 2014; Gkoutsioukosta and Apostolidou, 2023).

The sustainable integration of COMPUSEL into school curricula could enhance SEL, promote collaboration, and support innovative teaching practices. The alignment with Education for Sustainable Development (ESD) reinforces the need to develop students’ problem-solving and ethical decision-making skills (Bürgener and Barth, 2018; Cebrián et al., 2020; Mulà et al., 2017).

The study has some limitations that should be acknowledged. The short implementation period may have restricted the long-term development of SEL skills, as sustained interventions are often necessary to observe lasting behavioral and emotional changes. Additionally, the sample was limited to five countries, which affects the generalizability of the findings and highlights the need for broader, cross-cultural studies. Furthermore, cultural and methodological differences influenced the program’s outcomes, as variations in educational policies, teacher training, and school environments likely impacted the effectiveness of SEL interventions.

Future research should address these limitations by conducting longitudinal studies to assess the long-term retention of SEL skills and their impact over time. Expanding the sample to include more diverse educational contexts would also provide a clearer understanding of how SEL interventions can be adapted to different cultural and systemic realities. Additionally, component-specific analyses could help determine the distinct contributions of computational thinking and digital storytelling to SEL development. Finally, designing tailored SEL curricula that align with national policies and teacher training frameworks would enhance the effectiveness and sustainability of such programmes in different educational settings.

This proposal program offers an innovative and structured approach to SEL, integrating computational thinking and storytelling to equip students with emotional intelligence, problem-solving skills, and social responsibility. While results varied by country, the program’s potential for long-term educational benefits is evident. Strengthening teacher training, curriculum adaptation, and long-term assessments will be essential for ensuring its lasting impact in diverse educational contexts. As seen in the teacher interviews, the computational thinking framework encourages students to think systematically about social and environmental issues, fostering self-directed learning, participation, and collaboration (and in previous studies like Rieckman, 2018). Moreover, digital storytelling introduces students to ethical dilemmas and issues related to social responsibility, inclusion, and equity, reinforcing ESD principles.

Given the increasing emphasis on holistic education, it is essential to recognize that SEL and sustainability education are mutually reinforcing. SEL provides the emotional and cognitive foundations for students to engage with ethical decision-making, environmental responsibility, and global citizenship (Wals and Jickling, 2002). By integrating computational thinking into SEL instruction, COMPUSEL contributes to a more future-oriented, sustainable approach to education that prepares students for 21st-century challenges.

## 6 Conclusion

To conclude, this study reinforces a simple yet powerful idea: education is not just about knowledge; it’s about shaping individuals who can navigate life with confidence, empathy, and resilience. The COMPUSEL program has shown that integrating computational thinking and digital storytelling into social-emotional learning (SEL) can make a real difference in how students understand and manage their emotions, relate to others, and make thoughtful decisions.

Across different countries, we saw encouraging progress. In Greece, students gained greater self-awareness and self-management skills, while in Poland, relational skills and emotional regulation stood out. Turkey showed significant improvements in social awareness and collaboration, reflecting an increasing national focus on SEL. Portugal made strides in most areas, though responsible decision-making remains a challenge, and Romania’s limited changes suggest deeper structural factors at play. These variations remind us that education is deeply connected to culture, policies, and the realities of each learning environment.

Computational thinking emerged as a surprisingly effective tool, helping students break down emotional challenges into manageable steps, much like solving a puzzle. Digital storytelling, in turn, allowed them to engage with complex emotions in a safe and creative way, fostering empathy and deeper reflection. Teachers played a crucial role in this process, highlighting how these methods not only helped students navigate interpersonal conflicts but also encouraged collaboration, critical thinking, and problem-solving—skills that extend far beyond the classroom.

In summary, by embedding SEL in early education and leveraging innovative tools, we are not only enhancing academic learning but also preparing young people to engage meaningfully with the world. As global challenges grow more complex, we need individuals who can think critically, act responsibly, and care deeply about the impact they have on others and the planet.

Moving forward, it’s essential to refine and expand this exploratory experience, ensuring sustained teacher training, longer implementation periods, and deeper cultural adaptation. Research should continue to explore how SEL, computational thinking, and sustainability education intersect to equip students with lifelong skills.

To close, this study highlights a hopeful vision for the future of education—one that does not just teach students what to learn but helps them discover how to think, how to feel, and how to build a world that is not only smarter but also more compassionate and inclusive.

## Data Availability

The raw data supporting the conclusions of this article will be made available by the authors, without undue reservation.

## Footnotes

^1^ https://compuseleu.com/activity-book/

^2^ https://compuseleu.com/curriculum/

^3^ https://compuseleu.com/video-library/
